# The Antioxidant and Chemopreventive Activity of a Nutraceutical Derived from *Brassicaceae* Seed Extracts for Colorectal Cancer

**DOI:** 10.3390/nu17081358

**Published:** 2025-04-16

**Authors:** Ana Guzmán-Carrasco, Cristina Mesas, Kevin Doello, Jesús M. Porres, Alejandro García-Beltrán, Rosario Martínez, Francisco Bermúdez, Mercedes Peña, Consolación Melguizo, Jose Prados

**Affiliations:** 1 Instituto de Investigación Biosanitaria de Granada, ibs.GRANADA, 18012 Granada, Spain; anaguzman@correo.ugr.es (A.G.-C.); cristinam@ugr.es (C.M.); mpenacontreras@ugr.es (M.P.); jcprados@ugr.es (J.P.); 2 Department of Physiology, Institute of Nutrition and Food Technology (INyTA), Biomedical Research Center (CIBM), Sport and Health University Research Institute (IMUDS), Universidad de Granada, 18016 Granada, Spain; jmporres@ugr.es (J.M.P.); alejandrogb@ugr.es (A.G.-B.); rosariomz@ugr.es (R.M.); 3 Institute of Biopathology and Regenerative Medicine (IBIMER), Center of Biomedical Research (CIBM), University of Granada, 18100 Granada, Spain; 4 Medical Oncology Service, Virgen de las Nieves Hospital, 18016 Granada, Spain; 5 Department of Anatomy and Embryology, Faculty of Medicine, University of Granada, 18071 Granada, Spain; 6 Seed for Innovation S.L., Scientific Headquarters of the Almería Technology Park, Universidad de Almería, 04128 Almería, Spain; francisco.bermudez@beyond-seeds.com

**Keywords:** *Brassicaceae*, nutraceutical, *Eruca sativa*, *Sinapis alba*, colorectal cancer, cancer prevention

## Abstract

**Background.** Worldwide, colorectal cancer is the third most commonly diagnosed cancer. It is the second leading cause of cancer-related mortality. Recent studies establish a relationship between natural compounds from plants with the prevention and treatment of cancer. Specifically, glucosinolates with antitumoral capacity and polyphenols with the ability to scavenge free radicals that can cause cell damage have been identified in the *Brassicaceae* family. **Objectives**. Based on the previously mentioned factors, this study aimed to develop a nutraceutical made with extracts from different *Brassicaceae* seeds and study its antioxidant and antiproliferative action in vitro and in vivo using the AOM/DSS model in CC57BL6J mice. **Results**. Extract from the seeds of *Eruca sativa* and *Sinapis alba* showed the highest antioxidant capacity among the different species studied and were selected for nutraceutical formulation, which was potentially absorbable (73%) after an in vitro digestion process. In total, thirty compounds were identified in the nutraceutical that could be responsible for its antioxidant and tumoral prevention capacity. The intake of nutraceutical was a successful intervention to prevent the development of polyps by 31.6% and their size by 53.9%. When the nutritional intervention was used in combination with a physical exercise protocol, these parameters dropped to 52.3% and 62.6%, respectively. **Conclusions**. These findings suggest that the consumption of a diet rich in bioactive compounds from *Brassica* species, in combination with physical activity, is a valuable prevention strategy for colorectal cancer. However, more research is required to evaluate the efficacy and safety of these interventions in clinical settings.

## 1. Introduction

Colorectal cancer (CRC), defined as the abnormal and excessive proliferation of cells in the colon and rectum [1], is the third most common cancer worldwide. According to the World Health Organization (WHO), 1.93 million new cases of CRC were reported in 2020, affecting 1.07 million men and 865,630 women. It is also the second cause of cancer-related deaths, with 916,000 fatalities reported in 2022 (515,639 men and 419,536 women) [2]. The study published by Santucci et al. [3] evaluates cancer mortality data in the European Union from 1970–1974 to 2015–2019, showing a stronger decline from the year 2000 onwards. According to the prediction of these investigators, this trend is expected to continue. The predicted CRC mortality rate for 2024 (compared with data from 2015 to 2019) showed a decrease of 6.9% and 11.8% for men and women, respectively, considering all age groups. However, an increase in CRC-associated mortality is estimated in the 25–49 age group. Specifically, in Spain, an increase of 5.5% is expected in men, while in Italy, both sexes are affected. The sharpest increases for the predicted CRC mortality rate have been detected in the UK, with 26.1% in men and 38.6% in women. By 2040, the global mortality burden of CRC is expected to rise to 1.6 million deaths.

Risk factors for the development of CRC include (i) age, as it is diagnosed more frequently in individuals over 50 years old; (ii) predisposing diseases, especially the presence of intestinal polyps or inflammatory bowel disease; (iii) a history of CRC, since individuals with a previous CRC diagnosis are at higher risk of recurrence and metachronous intestinal tumors; (iv) genetic and familial factors, with approximately 25% of cases presenting a family history of CRC and 10% involving a hereditary component; (v) lifestyle factors, particularly physical inactivity; and (vi) dietary factors, which play an essential role and are under continuous research. In fact, this pathology has been associated with excessive alcohol intake, overweight and obesity, and certain types of food (e.g., processed meat) [2].

A consistent inverse association has been observed between the intake of legumes, vegetables, and fruits and the risk of cancer development. Thus, a higher consumption of vegetables is linked to a reduced cancer risk [2,4]. Recommended vegetables include carrots, green leafy vegetables, and vegetables of the genus Allium and the *Brassicaceae* family, among others. The recommendation of *Brassicaceae* consumption for CRC prevention is based on the content of molecules naturally present in this family known as glucosinolates, which are converted into bioactive products known as isothiocyanates (ITCs) through the action of the enzyme myrosinase and the intestinal microbiota. In addition, their high polyphenol content contributes to chemoprevention by scavenging free radicals that can cause cellular damage and enhancing the activity of detoxifying enzymes that inhibit the early stages of CRC development [5,6]. There are many factors that negatively affect the population’s direct intake of *Brassicacea* such as its characteristic bitter taste and smell [7]. This effect is mainly due to the presence of glucosinolates with a thiourea fraction which can negatively influence the preference in individuals more sensitive to this taste, an effect that is genetically determined [8,9]. In addition, these compounds can be altered and lose their functionality during cooking techniques involving high temperatures [10]. Specifically, between 20 and 40% of these compounds are lost during boiling [11].

However, despite their promising chemopreventive effects, the direct ingestion of *Brassicaceae* vegetables for further absorption and conversion into ITCs may not be sufficient to achieve therapeutic activity [12]. In this context, the use of nutraceuticals becomes an effective strategy in the prevention of CRC. Nutraceuticals are defined as food-derived products containing bioactive components that contribute to disease prevention and treatment. In oncology, these compounds have demonstrated significant efficacy in the prevention of tumor development by modulating the proliferation, differentiation, apoptosis, inflammation, angiogenesis, and metastasis of cancer cells. Moreover, their antioxidant properties may protect cells from free radicals that could cause DNA alterations [13,14]. Glucosinolates and phenolic compounds present in *Brassica* exert numerous health benefits, including antioxidant, anticancerogenic, antiaggregant, and detoxifying enzyme-inducing activities [15,16].

Based on the above-mentioned information, we hypothesize that a diet rich in bioactive compounds from *Brassica* species, combined with a structured physical exercise protocol, represents an effective therapeutic approach for CRC prevention. Additionally, dietary supplementation with a nutraceutical derived from *Brassica* seed extracts with high antioxidant capacity may help reduce oxidative stress and modulate gut microbiota. Therefore, this study aimed to (i) design a nutraceutical with high antioxidant and detoxifying enzyme-inducing capacity using different *Brassica* seed extracts, (ii) develop an animal experimental model of in situ CRC in mice, and (iii) assess the positive effects of a dietary intervention with the designed nutraceuticals and a mixed exercise program on oxidative stress, inflammation, and intestinal dysbiosis, aiming to expand our understanding of the molecular mechanisms underlying our experimental findings.

## 2. Materials and Methods

All procedures were carried out collaboratively between the Biomedical Research Center (CIBM) and the Sport and Health University Research Institute (IMUDS) of the University of Granada, Spain.

### 2.1. Plant Material

All the seeds that were analyzed in this study were supplied by the company Beyond Seeds S.L. (Almería, Spain). A total of six different plants from the *Brassicaceae* family were studied, including three different species, namely *Brassica oleracea*, *Eruca sativa* (*rocket*), and *Sinapis alba* (*mustard*), and three varieties of *Brassica oleracea*, *Brassica oleracea* var. *sabellica* (*Kale*), *Brassica oleracea* var. *italica* (*broccoli*), and *Brassica oleracea var. botrytis* (*cauliflower*).

### 2.2. Functional Extracts and Nutraceutical Formulation

Two distinct methods were employed to extract bioactive compounds from the plant material: (i) ethanolic extraction for the enhanced extraction of phenolic compounds and glucosinolates and (ii) the hydrolysis of proteins to produce bioactive peptides.

Ethanolic extraction: Seeds or freeze-dried plants were mixed with a hydroalcoholic solution (ethanol:water:HCl, 50:50:0.2 *v*/*v*/*v*) and extracted sequentially for 30 min under continuous stirring at 4 °C and at a pH of 2. This was followed by centrifugation (3500 rpm, 10 min), and the resulting pellet was reused for a new extraction procedure. All supernatants were pooled to form the final ethanolic extract, which was stored at –20 °C. To prevent oxidation, nitrogen (N_2_) was used following the method described by Kapravelou et al. [17].

Protein extraction and hydrolysis: Samples were mixed with distilled water (1:4) and extracted for 30 min under the following conditions: pH 8.8, T = 33 °C, and continuous stirred, as described by Kapravelou et al. [18]. After centrifugation (3500 rpm, 5 min), the pellet was subjected to a new extraction process, and combined supernatants were heated to 47 °C for 15 min, followed by the addition of 0.1 M CaCl_2_ and MgSO_4_ (1:100 *v*/*v*). Protein hydrolysis was conducted through the sequential addition of proteases from *Bacillus licheniformis* (0.3 AU/g protein) and *Aspergillus oryzae* (100 AU/g protein), each for 30 min at a pH of 8.8 and 47 °C.

Nutraceutical formulation: The nutraceutical was formulated by combining extracts to maximize their synergistic effects on oxidative stress, inflammation, detoxifying enzymes, and intestinal microbiome. The final selection was based on total polyphenol content, which was indicative of the antioxidant capacity. Because of differences in the freeze-drying behavior between ethanolic extracts and protein hydrolysates, the final formulation consisted of 10% ethanolic extract from *Eruca sativa* seeds, 10% from *Sinapis alba* seeds, 40% protein hydrolysate from *Eruca sativa*, and 40% from *Sinapis alba*.

The volumes of each extract were determined based on the yields after lyophilization (Cryodos-50, TELSTAR, Madrid, Spain). Prior to freezing, ethanol was removed from ethanolic extracts via vacuum evaporation (ThermoSci, Waltham, MA, USA).

### 2.3. In Vitro Digestion

The designed nutraceutical was subjected to an in vitro digestion process following the methodology described by Porres et al. [19], with slight modifications [20]. The process consisted of three phases: (i) gastric digestion for 2 h, (ii) pH equilibration for 30 min, and (iii) digestion and intestinal absorption through a 2 h equilibrium dialysis process (12,000–14,000 Da, Medicell Membranes Ltd., London, UK). To carry out the in vitro digestion process, 5 g of the sample was mixed with 100 mL of 0.01 N HCl, and the solution was adjusted to a pH of 2 using 1 N HCl. For gastric digestion, 20 mL of the digestion aliquot was mixed with 1 mL of the pepsin solution (0.16 g/mL in 0.1 N HCl). Following the initial preparation steps, dialysis membranes were introduced into the digestion vessels prior to pH adjustment using 0.1 N sodium bicarbonate (NaHCO_3_). Subsequently, 5 mL of 0.1 N NaHCO_3_ with bile salts (25 mg/mL) and pancreatin (4 mg/mL) was added to simulate intestinal digestion conditions. Upon completion of the digestion process, the contents inside the dialysis membranes, representing the dialyzed and potentially absorbable fraction, and those remaining outside, representing non-absorbed components potentially reaching the colon, were separately collected and stored at –20 °C for further analyses. Negative control assays were conducted using an equivalent volume of 0.01 N HCl. All procedures were carried out at 37 °C under constant agitation to mimic physiological conditions.

### 2.4. Antioxidant Activity

The quantification of total polyphenolic content was carried out via a modified Folin–Ciocalteu method according to Martínez et al. [21]. A gallic acid standard curve (0–600 µg/L) was constructed, and absorbance was measured at 760 nm using a Multiskan FC Microplate Photometer (Thermo Fisher Scientific, Waltham, MA, USA). The results were expressed as micrograms of gallic acid equivalents per milligram of extract (µg GAE/mg). A radical cation decolorization assay, ABTS [2,2′-azino-bis(3-ethylbenzothiazoline-6-sulfonic acid)], was assessed using the protocol established by Miller et al. [22]. Briefly, 6 µL of the extract or gallic acid standard solution (0–600 µg/L) was combined with 294 µL of the ABTS solution and incubated for 1–5 min. Absorbance was recorded at 620 nm, and the antioxidant capacity was expressed as µg GAE/mg of extract.

The Fe^2+^ chelating activity of the extracts was determined spectrophotometrically according to the method by Dinis et al. [23], while the reducing power (Fe^3+^ to Fe^2+^) was determined based on the procedures described by Oyaizu [24] and Duh et al. [25].

### 2.5. Chromatographic Studies via UPLC-QTOF Analysis

High-resolution mass spectrometric analysis of bioactive compounds in the nutraceutical formulation was performed using ultra-performance liquid chromatography coupled with quadrupole time-of-flight mass spectrometry (UPLC-QTOF; ACQUITY H CLASS and SYNAP G2, Waters, Milford, MA, USA). Prior to analysis, samples were filtered through 0.22 µm nylon syringe filters (Millipore, Burlington, MA, USA), and 10 µL aliquots were injected into the chromatographic system.

Phenolic compounds were separated using an ACQUITY HSS T3 analytical column (100 mm × 2.1 mm, 1.8 µm particle size), in accordance with the method detailed by Martínez et al. [26]. Mass spectrometry was performed under negative electrospray ionization (ESI^−^) mode by using high-purity nitrogen as both the desolvation gas (600 L/h) and the cone gas (30 L/h). Spectra were acquired across a mass-to-charge (*m*/*z*) range of 50–1200. Compound identification was tentatively performed based on retention times and characteristic mass fragmentation patterns using the MassLynx software v4.1 (Waters Corp., Milford, MA, USA).

### 2.6. In Vivo Experimental Design

#### 2.6.1. Animals

For the in vivo assay, 55 male C57BL/6J mice (body weight 15.3 ± 1.1 g at 4 weeks of age) were acquired from Charles River Laboratories (Barcelona, Spain). The mice were randomly assigned to four experimental groups and housed in groups of five per cage in a properly aerated and temperature-regulated space (21 ± 2 °C) (Animal Experimental Unit, CIC, University of Granada). The experiment lasted for 17 weeks, with the first week dedicated to allowing the animals to acclimate to their diet, housing, and training conditions. All animal procedures were conducted in accordance with the ethical guidelines for the care and use of laboratory animals and were approved by the Animal Experimentation Ethics Committee of the University of Granada, Spain (5/10/2021/148). The number of animals used (*n* = 15 for experimental group and 10 for CT−) was calculated following the principles of the 3Rs to ensure ethical research conduct [27]. It is worth noting that no mouse was excluded during the experimentation.

#### 2.6.2. Experimental Design

The experimental period lasted 17 weeks. Mice were randomly allocated to the following groups: CT−, the healthy control group that received a normocaloric standard diet (TD110675; 3.6 kcal/g, Teklad); CT+, which received a normocaloric standard diet and was subjected to CRC induction.; NT, which received a standard diet supplemented with 0.6% of the formulated nutraceutical following CRC induction; and NT + Ex, which received the same nutraceutical-supplemented diet as the NT group and underwent a structured training protocol during the whole experimental period. Animals were maintained under a reversed light/dark cycle (12:12 h), with ad libitum access to filtered water (type 2) and their respective diets. Daily food consumption was recorded, and body weight was measured weekly at a consistent time point. To ensure blinding, one of the authors was responsible for randomly assigning the experimental groups and providing the diet. At the conclusion of the experimental period, mice were anesthetized via intraperitoneal (IP) injection of ketamine (75 mg/kg) and xylazine (10 mg/kg). Organs were harvested, weighed, and examined for macroscopic abnormalities and immediately immersed in formaldehyde for histological processing. For colon tissue, the Swiss roll technique was used to facilitate the visualization of the intestinal epithelial morphology [28]. Additionally, a section of the colon was preserved in RNAlater (Ambion) and stored at –80 °C for subsequent RNA extraction.

#### 2.6.3. Tumor Induction in the Colon

Tumor induction was performed following the protocol described by Mesas et al. [29], with minor modifications. The development of a tumor was induced by an intraperitoneal injection of azoxymethane (Ref. A5486, Sigma-Aldrich, Madrid, Spain) at a dose of 12 mg/kg and three doses of 2% DSS (Ref. 42867-100MG, Sigma-Aldrich, Madrid, Spain) in drinking water.

#### 2.6.4. Exercise Protocol

To assess maximal aerobic capacity, an incremental treadmill running test was conducted to determine the maximal oxygen consumption (VO_2_ max) of the animals, following the protocol by Yang et al. [30], with minor modifications. Testing was conducted using a single-lane treadmill (Panlab LE8708) connected to an LE405 gas analyzer (Panlab, Harvard Apparatus, Barcelona, Spain). The initial treadmill speed of 20 cm/s was progressively increased by 3 cm/s every minute until a maximum speed of 100 cm/s was reached or until the animal reached exhaustion. For the exercise intervention, animals in the NT + Ex group were trained using a motorized rodent treadmill (Panlab LE8710RTS). During the first week, mice underwent an adaptation period consisting of 10 min of treadmill running per day at 12 cm/s on a 0° incline. This was followed by a structured training program developed by our research group (see Table 1). The protocol consisted of moderate-to-high intensity aerobic exercise performed 5 days per week during the animals’ dark cycle, with each session lasting 25 min. In brief, the protocol included a 5 min warm-up at 20% VO_2_ max, followed by a first moderate-intensity cycle that consisted of 1.5 min of a progressive speed increase from 30 to 45% VO_2_ max and 1 min at 45% VO_2_ max. This was followed by four moderate-intensity sets in which the speed of 45% VO_2_ max was maintained for 2 min, with additional intervals at 30% VO_2_ max for 1 min and 1.5 min rest.

#### 2.6.5. Morphological and Histological Analysis

The colons of the mice were excised, opened, and photographed using the ImageJ software v1.54d for polyp counting and measurement. The fixation of both organs and Swiss roll sections was performed in paraformaldehyde (24 h) and embedded in paraffin blocks. Subsequently, 5 µm sections were cut on a rotary microtome (Leica, Wetzlar, Germany). Finally, the sections were deparaffinized, hydrated, and stained with hematoxylin and eosin (H&E).

#### 2.6.6. Gene Expression Assays

After homogenizing the colon aliquots, total RNA was extracted using 1 mL of Tri-Reagent (Sigma-Aldrich), solubilized in RNase-free water, and treated with DNase (Applied Biosystems, Waltham, MA, USA) to eliminate any DNA. Reverse transcription of RNA (100–250 ng) was then performed in a Lifepro thermal cycler (Bioer Serves Life, Hangzhou, China) according to standard protocols. Quantitative RT-PCR was performed with the Quantum Studio 12 K Flex Real-Time PCR System using primers (Applied Biosystems) for genes involved in oxidative metabolism, detoxification pathways, inflammatory processes, or in tumor development (Table 2). For the PCR reaction, the master mix consisted of the first-strand cDNA template, primers, 2X TaqMan Fast Universal PCR Master Mix, and No AmpErase UNG (Applied Biosystems). *ß-actin* was used as an internal control. Data analysis was performed using the 2 ΔΔCt method with the control group as the reference.

#### 2.6.7. Metagenomic Analysis

Cecal contents were collected for the isolation of genomic DNA (gDNA). Extraction was performed using the QIAamp^®^ (Venlo, The Netherlands) PowerFecal^®^ (Woodcliff Lake, NJ, USA) DNA kit according to the manufacturer’s protocol for process automation using the QIAcube robot. The concentration of gDNA was determined using fluorometry (qubit). The V4 region (233 bp) of 16S ribosomal RNA (rRNA) genes was sequenced by the MiSeq system (Illumina, San Diego, CA, USA) for the analysis of gDNA samples. The Institute of Parasitology and Biomedicine López-Neyra (IPBLN) of the Spanish National Research Council (CSIC, Granada, Spain) performed the library preparation, pooling, and miniSeq sequencing.

### 2.7. Statistical Analysis

Descriptive statistical methods were employed, like absolute frequency statistics which are expressed as the mean and standard deviation and relative frequency ones which are expressed as percentages. Significant differences were analyzed using one-way analysis of variance (ANOVA), and Tukey’s test was used to distinguish differences between means. Results are reported as the mean plus standard deviation (SD) of three replicates for in vitro experiments and of ten (the CT− group) and fifteen (CT+, NT, and NT + Ex groups) replicates for the in vivo study. Eight animals were included in the gene expression analysis. For in vitro digestion results, student’s *t*-tests were applied to analyze significant differences. Statistical Package for Social Sciences (IBM SPSS for Windows^®^, version 22.0, Armonk, NY, USA) was used for all the analyses, and the significance level was set at *p* < 0.05.

## 3. Results

### 3.1. Antioxidant Capacity of Brassicaceae Extracts

The extraction yield and antioxidant capacity of the different functional extracts obtained from *Brassicaceae* seeds, as well as the nutraceutical formulated, are shown in Table 3. Regarding the ethanolic extracts, *Sinapis alba* seeds showed a higher extraction yield (120.8 ± 1.3 mg of extract per gram of flour), as well as a significantly higher concentration of total polyphenols (60.5 ± 1.0 µg gallic acid equivalent per mg of extract). The ethanolic extract of *Eruca sativa* seeds demonstrated the highest antioxidant capacity in the rest of the tests. Specifically, in the ABTS test and iron-reducing capacity assay, it achieved values of 15.0 ± 0.0 and 43.5 ± 1.4 µg gallic acid equivalent per mg of extract, respectively, while its iron-chelating activity was 3.99 ± 0.06 chelating activity units per mg of extract (CAU/mg).

A similar trend of results was obtained from the protein hydrosylates. The protein hydrolysate from *Eruca sativa* seeds exhibited significantly higher values than the other species tested in their ability to scavenge ABTS free radicals (17.0 ± 0.4 µg gallic acid/mg hydrolysate) and their iron-chelating activity (1.34 ± 0.02 CAU/mg HP), whereas the protein hydrolysate from *Sinapis alba* seeds yielded the highest extraction efficiency (31.9 ± 1.1%). In this case, the highest concentration of total polyphenols was observed in the hydrolysates of both seeds, with 33.6 ± 0.4, and 31.7 ± 0.4 µg gallic acid/mg hydrolysate for *Eruca sativa* and *Sinapis alba*, respectively. Based on these findings, a nutraceutical was developed using these two plant species, which maintained the antioxidant capacity in all chemical assays (Table 3).

### 3.2. In Vitro Digestion

The results of the in vitro digestion tests are summarized in Table 4. After the digestion process, 73.1% of the formulated nutraceutical was found to be potentially absorbable. Additionally, in vitro digestion did not affect the antioxidant capacity of either the bioavailable (dialyzed) fraction or the non-absorbable components (retained) that reach the colon. In both cases, the antioxidant capacity remained significantly higher than that of the blank.

### 3.3. Mass Spectrometry Identification of Bioactive Compounds

**Figure 1:**
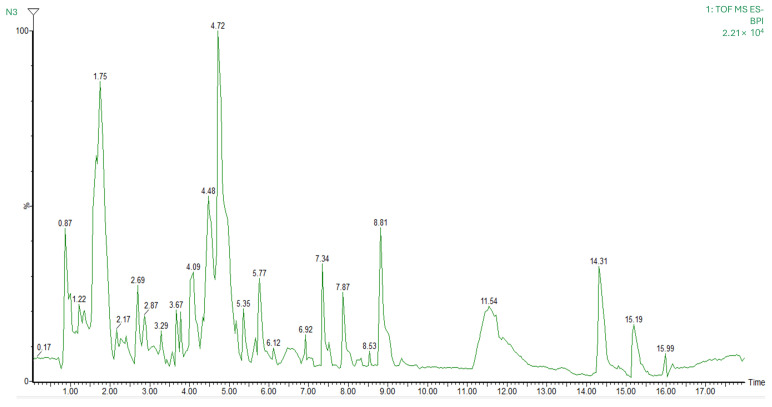
Chromatographic profile of bioactive compounds identified in the nutraceutical using high-performance liquid chromatography.

The chromatographic profile and the main compounds tentatively identified in the nutraceutical formulation are presented in Figure 1 and Table 5, respectively. A total of 30 biocompounds were identified, with flavonoids being the most representative class including 10 compounds (cucumerin B, dichamanetin, epigallocatechin, eucalyptin, gallocatechin, moracenin D, nobiletin, pectolinarigenin, prenylnaringenin, and quercetin), followed by terpenoids with 6 compounds (chamissonolide, hydrangenoside A, kirenol, ligustrosidic acid, lucidumoside C, and pyrohyperforin). In addition, two compounds were identified within the phenolic acid class (curculigoside A and populin), as well as two phenilpropanoids (atharticin and nothoapiole) and two glucosids (marinoid D and prunioside A). Other identified compounds belonged to the following groups: alkaloid (nesodine), chromones (cimifugin), quinone (rubialatin A), isoflavans, furans (mumefural), isoflavones (glycitin), ether (crotepoxide), long-chain fatty alcohol (persealide), and lignans (diphyllin).

### 3.4. In Vivo Experimental Model

#### 3.4.1. Body Weight and Food/Water Intake

**Figure 2:**
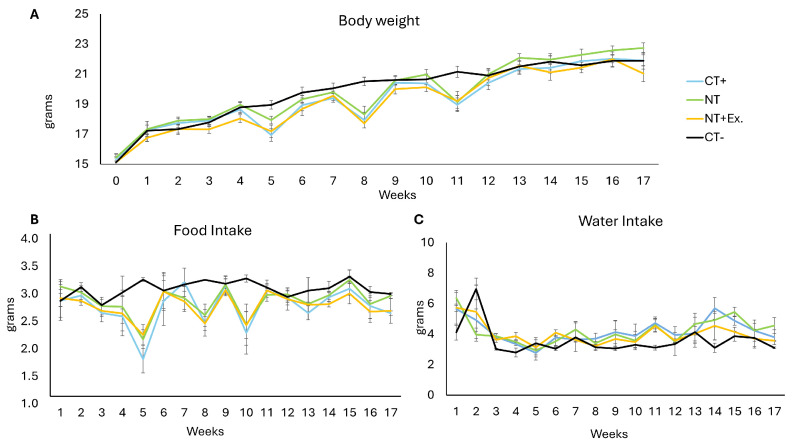
(**A**) Body weight, (**B**) food intake, and (**C**) water intake of mice fed different experimental diets. CT−, healthy animals fed the control diet. CT+, tumor-induced animals fed the control diet. NT, tumor-induced animals fed the control diet supplemented with the nutraceutical. NT + Ex, tumor-induced animals fed the control diet supplemented with the nutraceutical combined with the exercise protocol. Results are expressed as the means plus the SD (vertical bars) of 15 replicates.

Figure 2 illustrates the effects of colon cancer induction and interventions on body weight and food/water intake in mice throughout the experimental period. The initial animal weight was 15 ± 1 g, and all animals reached 22 ± 2 g by the end of the study period (Figure 2A). A temporary weight loss was observed at weeks 5, 8, and 11 in the CT+, NT, and NT + Ex groups, corresponding to the administration of DSS in drinking water for tumor induction. Regarding food intake (Figure 2B), no significant differences were observed between groups, except at weeks 5, 8, and 10, when a decrease in intake was detected in the tumor-bearing groups (CT+, NT, and NT + Ex) due to DSS-induced discomfort. However, there were no significant differences in the intake of drinking water when the DSS was administered (Figure 2C).

#### 3.4.2. Tissue and Organ Weights

The effects of tumor development and the different interventions performed on organ weight and colon length are presented in Table 6. Tumor development led to a significant increase in the weight of the colon, spleen, and liver, although the increment in liver weight was not statistically significant. In addition, tumor development led to a shortening of the colon and the thickening of its wall. Only the nutritional intervention (NT group) significantly reduced colon weight (0.25 ± 0.02 g) and prevented colon shortening, with values comparable to the healthy control group (6.28 ± 0.15 cm vs. 6.88 ± 0.18 cm, respectively).

#### 3.4.3. Modulation of Polyp Size and Number

**Figure 3:**
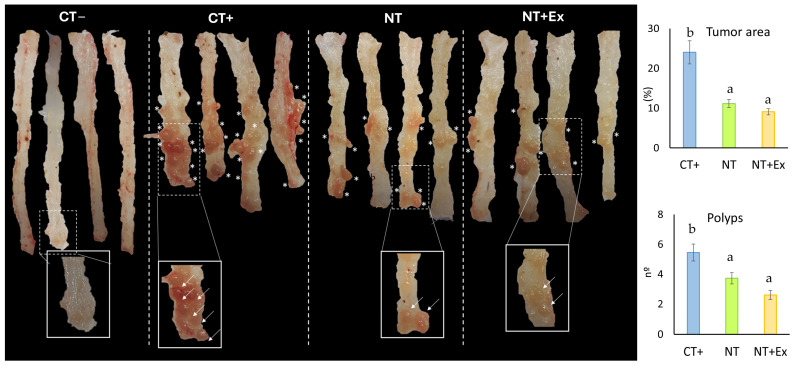
Effect of the nutritional intervention (NT), alone or in combination with physical exercise (NT + Ex), on the number of polyps and the area occupied by polyps. Asterisks (*) indicate each of the polyps identified in the colons of the animals. Arrows point to the polyps located in the enlarged segment of the colon. Results are expressed as the means plus the SD (parenthesis) of 15 replicates. Means values with different letters (a and b) represent significant differences (ANOVA, *p* < 0.05).

The effect of the nutritional intervention, alone or combined with the physical exercise protocol, on the number of polyps and the area occupied by these polyps is shown in Figure 3. Tumor induction resulted in polyp development in the positive control group (CT+) of 24.3 ± 2.9% of the total colon area. In the NT group, polyps were significantly smaller, occupying 11.2 ± 1.0% of the total colon area. Although not statistically significant, the area occupied by the tumor was further reduced to 9.1 ± 0.8% when the nutraceutical was combined with the exercise program (NT + Ex). Regarding the number of polyps, the CT+ group showed a mean of 5.45 ± 0.52 polyps, whereas the NT group significantly reduced this number to 3.73 ± 0.38 polyps. In the NT + Ex group, polyps were further reduced to 2.60 ± 0.31.

#### 3.4.4. Histological Analysis

**Figure 4:**
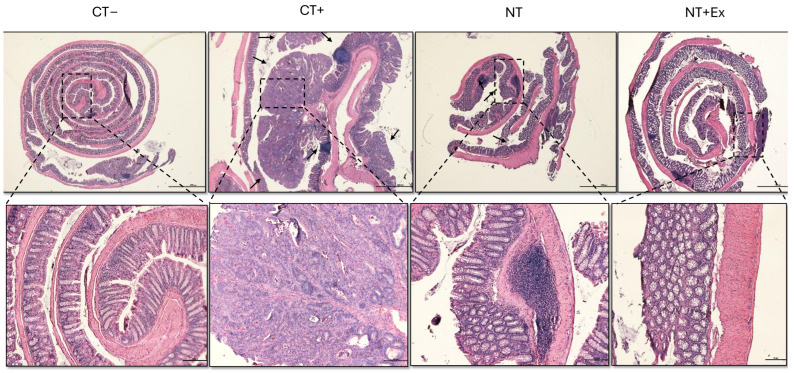
Representative H&E-stained histological images following tumor induction via AOM/DSS. The Swiss roll technique was used to analyze the colon (scale bar = 1000 μm [top row] and 100 μm [bottom row]). Arrows indicate polyps with dysplasia. CT−, healthy animals fed the control diet. CT+, tumor-induced animals fed the control diet. NT, tumor-induced animals fed the control diet supplemented with the nutraceutical. NT + Ex, tumor-induced animals fed the control diet supplemented with the nutraceutical combined with the exercise protocol.

**Figure 5:**
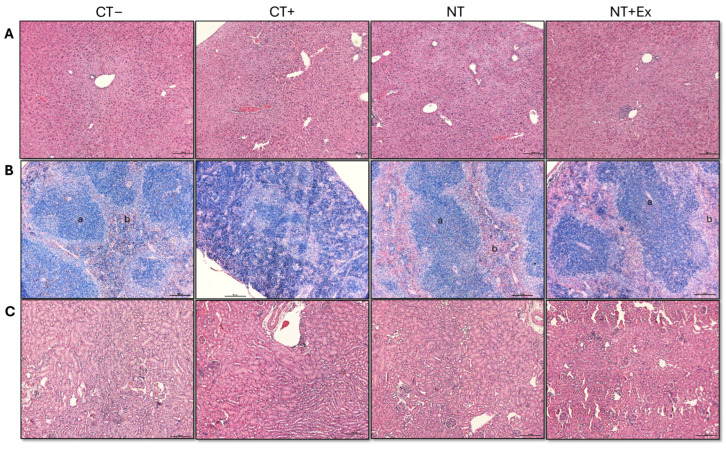
Representative images of the histological analysis of organs from different experimental groups. The H&E method was used to analyze the liver (**A**), spleen (**B**), and kidneys (**C**). In spleen samples, the white pulp and the red pulp are indicated by (a) and (b), respectively. Bars, 100 µm.

The histological analyses using H&E staining of samples from the Swiss Roll colon, liver, spleen, and kidney are shown in Figure 4 and Figure 5. The positive control (CT+) exhibited disrupted colonic crypt architecture, the absence of goblet cells, and dysplastic polyps marked by colonic epithelial cells with abnormal nuclei. In contrast, the Swiss roll colon samples from treated mice (NT and NT + Ex) showed a significantly lower number of polyps, and those that developed polyps exhibited a lower degree of dysplasia compared to those in the untreated group (CT+). In the CT+ group, a disruption in the organization between the white pulp and the red pulp of the spleen was also observed. The groups that ingested the nutraceutical throughout the experimental period were able to reverse this effect, showing an organization very similar to that of the healthy control group.

#### 3.4.5. Gene Expression

Table 7 presents the gene expression levels in the colon of experimental animals. Tumor induction and development led to a significant increase in the expression of the *slc20*, *pik3cd*, and *IL-1b* genes, whereas the expression of *gpx2*, *sod1*, *gsta1*, and *IL-6* was reduced. The different interventions successfully restored the expression levels of *gpx2*, *sod1*, *pik3cd*, and *slc20* to normal values. Moreover, they significantly increased the expression of *noq1* and decreased the expression of cadm1, *cdc42*, and *IL-6*, even below the levels observed in the control group.

#### 3.4.6. Metagenomic Analysis

**Figure 6:**
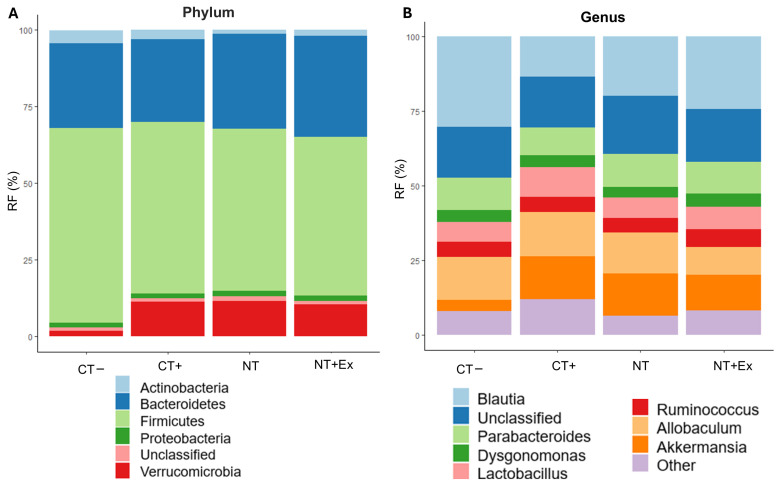
Metagenomic analysis of caecum content in the experimental groups, showing bacterial abundance at the level of phylum (**A**) and genus (**B**). CT−, healthy animals fed the control diet. CT+, tumor-induced animals fed the control diet. NT, tumor-induced animals fed the control diet supplemented with the nutraceutical. NT + Ex, tumor-induced animals fed the control diet supplemented with the nutraceutical combined with the exercise protocol. Results are the means of 8 replicates.

Figure 6 illustrates the effect of tumor development, nutritional interventions, and physical exercise on the relative abundance of bacteria in the cecum content of the experimental groups. Tumor development resulted in a significant increase in the phylum *Verrucomicrobia* that was not reversed with the different interventions applied. No additional significant differences related to tumor development were observed, but the presence of bacteria belonging to the phylum *Firmicutes* and *Actinobacteria* was reduced in the groups that had developed a tumor and had been fed the nutraceutical, while the presence of *Bacteroidetes* was increased in the group that also underwent the physical exercise protocol. Consequently, the ratio of Firmicutes/Bacteroidetes was significantly decreased by the nutritional intervention. The development of CRC significantly increased the presence of bacteria belonging to the *Verricomicrobiaceae* family and other unclassified families and decreased that of *Lachnospiraceae* and *Porphyromonadaceae*. These last two were reverted to the standard values of healthy mice when the nutraceutical was administered in the diet and the physical exercise protocol was performed.

At the genus level, *Blautia* was significantly reduced due to tumor development. However, its concentration increased with the nutritional intervention treatment, reaching levels comparable to those of healthy mice (CT−) when combined with the physical exercise protocol (NT + Ex). The *Lactobacillus* genus significantly increased its concentration with tumor development and was influenced by the nutritional intervention, showing similar values to the control group, that is, those animals that had been fed the nutraceutical (NT). The presence of the genus *Akkermansia* showed a significant increase in all groups that had developed the tumor.

## 4. Discussion

Natural products are a promising tool for treating or preventing CRC. Nearly 50% of the currently available treatments for CRC have been developed directly or indirectly from natural compounds [31,32]. Among these, the *Brassicaceae* family has been identified as an excellent source of bioactive compounds with antitumor properties [33]. Of all the *Brassicaceae* extracts analyzed in this study, the highest extraction yield and the highest polyphenol content were obtained in the ethanolic extract of *Sinapis alba* seeds. For ABTS, lipid peroxidation inhibition, and iron-chelating or iron-reducing capacity tests, the best results were achieved with the *Eruca sativa* seed extract. These results are consistent with the findings by Khalil et al. [34], who reported that *Eruca sativa* seeds showed a significantly enhanced ability to scavenge 2,2-diphenyl-1-picrylhydrazyl (DPPH) free radicals in comparison to other *Brassicaceae* species.

To induce in situ CRC in C57BL-6J mice, an intraperitoneal administration of AOM and three administrations of DSS in the drinking water were employed. This CRC induction model has been successfully validated in previous studies from our research group [29] and is widely used in the study of CRC, as it accurately replicates the full process of colon carcinogenesis, from the initial crypt proliferation phase to the final development of carcinoma [35]. Specifically, as we demonstrate in histological studies, the induction by AOM and DSS induces dysplastic polyps that are the previous step to invasive carcinoma, as described by Robertis et al. [36] AOM/DSS-induced tumors have mutations of Kas and β-catenin but not p53 mutations.

The nutritional intervention, either on its own or combined with the training protocol, significantly reduced the number of polyps developed in the colon with respect to the untreated control group (CT+). This effect can be attributed to the bioactive compounds identified in the nutraceutical, as in vitro digestibility tests confirmed that dialysates and retained fractions from nutraceutical-enriched diets exhibited higher antioxidant activity than those from the control diet. These findings suggest that some of the antioxidant compounds in the nutraceutical were potentially absorbable, which facilitates the exertion of beneficial systemic effects [37,38]. Specifically, several bioactive molecules identified in the nutraceutical have been previously studied for their anticancer properties in CRC. The flavonoid pectolinarigenin has demonstrated anticancer effects both in vitro and in vivo by inhibiting the cell growth, migration, and invasion of colon cancer cells (HCT116 and CT26). Furthermore, it has been shown to reduce the size and number of tumor nodules in a subcutaneous cancer model of Balb/c mice induced with CT26 colon cancer cells [39], T24 bladder cancer cells [40], or ACS gastric cancer cells [41]. Other authors have reported that the flavonoid epigallocatechin is able to inhibit tumor cell growth via the PI3K pathway and can be used as a chemotherapeutic [42], a chemopreventive agent [43], or as combinatorial therapy with anti-CTLA4 [44] against CRC. Dichamentin has shown a cytotoxic effect against HCT-116 CRC cells while demonstrating no cytotoxic effects on the non-cancerous lung cell line MRC-5 [45]. Quercetin exerts chemopreventive effects by inducing a large number of transcription factors that regulate metabolic pathways such as cell cycle, cell adhesion, gene transcription, or the immune response [46]. Nobiletin is another flavonoid with preventive effects on CRC progression, which is mediated by modulating the Akt signaling pathway, suppressing angiogenesis, and inhibiting CRC progression [47].

The preventive effect of *Brassicaceae* consumption against tumor development has been demonstrated in several studies. For instance, Baenas et al. [48] reported that the administration of 100 mg/kg of an aqueous extract from broccoli sprouts 30 days before tumor induction significantly reduced brain tumor size and progression in an experimental rat model. Similarly, *Brassicaceae* consumption has been shown to reduce preneoplastic colon lesions as well as the expression of cancer stem cell markers in rats [49]. This effect has been attributed to the set of phenolic compounds, isothiocyanates, vitamins, and nutrients present in these species, which act as an effective and favorable matrix in the stimulation of detoxifying enzymes and activators of the cellular antioxidant defense process [48]. Several population-based studies have examined the relationship between *Brassica* consumption and the decrease in the risk of developing CRC [50]. In the study by Mori et al. [51], no significant association was found between *Brassica* consumption and the risk of developing CRC in the Japanese population. However, these authors identified a positive association between plant-derived polyphenol consumption and CRC prevention, which is in agreement with our findings.

In the present study, no metabolic alterations or organ lesions derived from nutraceutical ingestion were found. In fact, the null toxicity of ethanolic extracts of *Eruca sativa* and *Sinapis alba* seeds has been reported by other authors. For instance, Xian et al. [52] demonstrated that the ingestion of up to 2500 mg/kg (maximum tested dose) of an ethanolic extract of *Sinapis alba* seeds proved to be innocuous in an experimental animal model of Balb/c mice, showing no signs of toxicity, deterioration, or death in the animals. Similarly, the nasogastric administration of up to 200 mg/kg of an ethanolic extract of *Eruca sativa* seeds also showed no signs of toxicity in an experimental Wistar rat model [53].

CRC is associated with oxidative stress and chronic inflammation [54,55]. In this experiment, tumor development led to a reduced expression of antioxidant enzymes (*gpx* and *sod1*) and the detoxification enzyme *gst*, along with an increased expression of transcription factors related to inflammatory activity such as *IL-1ß*. All these parameters were reversed in the groups that ingested the nutraceutical. Furthermore, the former intervention increased the transcript expression of quinone reductase enzyme (*nqo1*), which is involved in cellular detoxification, and decreased the expression of *IL-6*. These effects are assignable to the high phenolic content of the nutraceutical, as phenolic compounds are potent antioxidants capable of neutralizing free radicals; preventing the oxidation of lipids, proteins, and nucleic acids; and reducing colonic mucosa inflammation [56]. Other authors have reported that the administration of 200 mg/kg of an ethanolic extract from *Sinapis alba* seeds reduced the mRNA expression of *TNFα*, *IL-1ß*, and *IL-6* in the colon of Balb/c mice [52]. Similarly, the ingestion of 200 mg/kg of an ethanolic extract from *Eruca sativa* seeds increased the activity of the antioxidant enzymes sod and gpx in the testes of Wistar rats exposed to acrylamide-induced oxidative stress [53]. Therefore, these compounds may reduce the risk of exposure and susceptibility to carcinogenesis [5]. In addition to their previously reported antiproliferative in vitro and in vivo effects, flavonoids play a pivotal role in tumor prevention by modulating oxidative stress and chronic inflammation. As discussed earlier, reactive oxygen species (ROS) can activate transcription factors involved in tumor generation and progression by increasing genomic instability, cell proliferation, and the risk of DNA damage or mutation. Furthermore, the tumor microenvironment is highly oxidative, which inhibits the activity of endogenous antioxidant enzymes such as glutathione peroxidase. Therefore, the antioxidant capacity conferred by these molecules is essential for counteracting these tumor-promoting factors [14,57].

As described by Mesas et al. [29], the effect in the prevention of new dysplastic polyps in colonic mucosa could be related to the anti-inflammatory effect of the extract that could reduce ROS production and the mutagenesis related to polyp formation.

Tumor development increased *pik3cd* and *slc20a1* expression with respect to the healthy control group. The overexpression of *pik3cd* correlates with increased *β-catenin* levels, which translates to increased signaling of the Wnt/β-catenin pathway, which is involved in CRC initiation and progression [58]. Although *Slc20a1* is not a specific CRC marker, its overexpression has been related with a poor prognosis of prostate cancer [59] and breast cancer [60]. Moreover, *Slc20a1* is involved in Wnt/β-catenin signaling pathway activation [61]. The expression of these markers was reversed following the different interventions implemented, with the effect being more pronounced in the group combining both interventions (NT + Ex). *Cdc42* is crucial in tumor generation, invasion, and metastasis because of its role in essential physiological processes, including cytoskeleton and microtubule regulation, transcription, cell cycle progression, and apoptosis [62]. Therefore, its overexpression is strongly associated with CRC development [63]. However, in our study, *cdc42* expression did not show significant differences with tumor development, although its expression was reduced in animals that receive the nutritional intervention, either alone or combined with physical exercise. Similar results were reported in a study by Guo et al. [13], where tumor development in AOM/DSS-induced CRC in C57BL/6 mice led to an increase in *pik3cd* and *cdc42* expression, whereas it was reduced in the group of mice that ingested 2% curcumin for 12 weeks.

The significant impact of nutraceutical administration on the microbiome under our experimental conditions can be clarified by the large percentage of ingested flavonoids that are not absorbed in the small intestine and reach the colon directly, where they interact with the microbiota, stimulating the production of short-chain fatty acids and the proliferation of beneficial bacteria [64]. The composition of the microbiome in animals that have developed CRC revealed an increased concentration of the phylum Verrucomicrobia, whereas the relative abundance of the phylum Firmicutes and Actinobacteria was significantly lower compared to the healthy control group (CT−). These findings are consistent with those reported by Hidalgo-García et al. and Song et al. [65,66]. However, while these authors also observed an increase in the phylum Bacteroidetes, such a shift was not detected in our study. These abnormal changes in microbiome composition may involve the destruction of the intestinal barrier by increasing the inflammatory response and damage to the gut mucosa [65,67]. The observed decrease in Firmicutes abundance could be due to the pro-inflammatory state associated with tumor induction, as this CRC model is characterized by inflammation-driven tumorigenesis [66,68].

The Firmicutes/Bacteroidetes (F/B) ratio is typically reduced in CRC development and tends to increase following dietary or therapeutic interventions [65,69,70]. This effect may be linked to an increase in Firmicutes, which are associated with butyrate production, a short-chain fatty acid that has been shown to induce apoptosis in colon cancer cells, reduce inflammation, and preserve intestinal mucosal integrity [71]. Under our experimental conditions, the F/B ratio was reduced following CRC induction and further decreased after the nutraceutical and exercise interventions. However, it is important to note that while the F/B ratio is often used as a biomarker for gut dysbiosis, its interpretation remains controversial, as its significance may vary across different pathological conditions [66,72].

As previously mentioned, none of the interventions carried out were able to reverse these alterations. Conversely, an increase in the abundance of Bateroidetes, a phylum associated with CRC susceptibility and promotion [71], was observed. However, since Bacteroidetes was not represented at the genus level, it is unlikely that there was an increased presence of harmful species such as Bacteroides fragilis, a key contributor to CRC development due to its ability to induce inflammation and alter gut ecology, thereby facilitating the colonization of pathogenic bacteria such as *E. coli* and *Fusobacterium nucleatum* [73]. In fact, under our experimental conditions, the presence of *Blautia* (phylum *Bacillota*, family *Lachnospiraceae*) was increased compared to CT+. *Blautia* has been reported to inhibit CRC progression by enhancing CD8+ immune cell activity [74]. Similarly, we detected an increase in *Akkermansia* (phylum *Verrucomicrobia*, family *Verrucomicrobiaceae*), specifically *Akkermansia muciniphila*, which has been shown to protect against CRC development in murine models by inhibiting the *AhR/β*-catenin signaling pathway [75], in addition to promoting the release of extracellular vesicles that have a multitude of regulatory effects on health, including improved mucosal integrity, reduced intestinal permeability, and an increase in beneficial bacteria [76]. Consequently, this genus is associated with a healthy microbiome that can protect against CRC development [65]. However, to establish a definitive link between microbiome modulation and CRC prevention, further studies are needed to identify the abundance and composition of microbial populations in precancerous tissues [73].

The gut microbiome can exert a direct influence on the organism’s oxidative and inflammatory status. Specifically, microorganisms can activate the host’s antioxidant defense mechanisms or exert their effects by activating their own antioxidant enzymes, including superoxide dismutase (SOD) and catalase. Additionally, they produce metabolites such as short-chain fatty acids (SCFAs), which can regulate the organism’s oxidative status. Specifically, SCFAs (acetate, propionate, and butyrate) induce the activity of the transcription factor nuclear factor erythroid 2-related factor 2 (Nrf2) in epithelial cells, which regulates the expression of antioxidant and detoxifying enzymes, thereby enhancing antioxidant activity [77]. Any disruption of the microbiome may lead to the proliferation of pro-oxidant bacteria such as Listeria and Clostridium, which can impair epithelial cell metabolic function by producing hydrogen sulfide (H_2_S) through the degradation of sulfur-containing amino acids. This metabolite inhibits the mitochondrial electron transport chain in epithelial cells, thereby promoting oxidative stress.

It is well-established that an imbalance in oxidative status triggers the expression of pro-inflammatory cytokines. These changes increase membrane permeability, facilitating the translocation of bacteria into the lamina propria and further amplifying ROS production and the inflammatory response. Sustained disruption of this balance over time contributes to the development of colitis and colorectal cancer [77].

The limitations of this study are that the AOM/DSS model for polyp development will be affected by the type of mouse strain used, since the mouse strains used present different sensitivity to the treatment. Another limitation is the impossibility to control the daily intake of DSS consumed by each mouse. Therefore, we propose to experimenters who intend to reproduce the study in the future to evaluate the dose of AOM/DSS to be used in their study in a previous trial to minimize environmental variations in their trial and to place an automatic water system, if possible. Another factor to consider is that animals during treatment with AOM/DSS can lose a significant amount of weight, so we recommend weighing the animals daily (during DSS intake and one week after), and in case of marked weight loss and weakness, administer 1mL of saline solution via IP.

## 5. Conclusions

The outcomes of this study reveal the beneficial effects of *Brassicaceae* extracts on health and support their potential use as dietary supplements for the prevention of pathological conditions in which oxidative stress plays a key role. Bioactive compounds derived from this family could be a key element in reducing the risk and incidence of CRC by scavenging unstable molecules such as reactive oxygen species (ROS), which can initiate the process of carcinogenesis. The intake of a *Brassica* seed-based nutraceutical proved to be an effective intervention, reducing polyp development by 31.6% and polyp size by 53.9%, this effect was increased to 52.3% and 62.6%, respectively, when the nutritional intervention was combined with a physical exercise protocol. Despite the promising results of this study, further trials are needed to define whether similar results can be achieved in other animal models and to transfer their potential applicability to CRC prevention in humans.

## Figures and Tables

**Table 1:** Training protocol.

Phase	Time	% VO_2_ Max
Warm-up	5′	20
Exercise program		
1×	1′30″	20 → 45
	1′	45
4×	2′	45
	1′	30
	1′30″	Rest

**Table 2:** Reference of primers for genes analyzed in the study.

Function	Genes	Primers
Oxidative metabolism	*cat*	Mm00437992
	*gpx2*	Mm01286848
	*sod1*	Mm01344233_g1
Detoxification pathways	*nqo1*	Mm00500822_g1
	*gsta1*	Mm03019257_g1
Inflammatory process	*IL-1b*	Mm00434228_m1
	*IL-6*	Mm00446190_m1
Tumor development	*cadm1*	Mm00457551_m1
	*cdc42*	Mm01194005_g1
	*pik3cd*	Mm00435674_m1
	*slc20*	Mm00489378_m1

**Table 3:** Antioxidant capacity of the different *Brassicaceae* seed extracts and the nutraceutical.

	Yield (mg/g)	TPC (µg GA)	ABTS (µg GA)	ICC (CAU)	IRC (µg GA)
EtOH					
*BO* var. *sabellica*	115.4 ± 2.9 ^b^	30.9 ± 0.5 ^b^	11.6 ± 0.4 ^bc^	1.56 ± 0.11 ^b^	25.9 ± 1.4 ^b^
*BO* var. *italica*	115.3 ± 9.7 ^b^	31.4 ± 0.9 ^b^	12.8 ± 0.1 ^c^	1.29 ± 0.09 ^b^	27.7 ± 0.3 ^b^
*BO* var. *botrytis*	132.1 ± 4.6 ^b^	27.9 ± 0.4 ^a^	10.2 ± 0.7 ^b^	0.87 ± 0.01 ^a^	20.1 ± 0.6 ^a^
*Eruca sativa*	86.9 ± 1.9 ^a^	43.5 ± 0.4 ^c^	15.0 ± 0.0 ^d^	3.99 ± 0.06 ^c^	43.5 ± 1.4 ^c^
*Sinapis alba*	120.8 ± 1.3 ^b^	60.5 ± 1.0 ^d^	7.4 ± 0.1 ^a^	0.98 ± 0.05 ^a^	18.8 ± 1.2 ^a^
HP					
*BO* var. *sabellica*	24.4 ± 0.2 ^b^	28.5 ± 0.3 ^b^	12.2 ± 0.7 ^a^	1.00 ± 0.03 ^a^	13.1 ± 0.3 ^b^
*BO* var. *italica*	27.3 ± 0.3 ^c^	26.2 ± 0.3 ^ab^	10.2 ± 0.3 ^a^	1.10 ± 0.02 ^ab^	12.9 ± 0.2 ^b^
*BO* var. *botrytis*	29.3 ± 0.6 ^cd^	25.1 ± 1.1 ^a^	11.5 ± 0.7 ^a^	1.19 ± 0.07 ^b^	13.0 ± 0.2 ^b^
*Eruca sativa*	20.3 ± 0.4 ^a^	33.6 ± 0.4 ^c^	17.0 ± 0.4 ^b^	1.34 ± 0.02 ^c^	12.8 ± 0.1 ^b^
*Sinapis alba*	31.9 ± 1.1 ^d^	31.7 ± 0.4 ^c^	11.1 ± 0.4 ^a^	0.98 ± 0.02 ^a^	8.0 ± 0.1 ^a^
NT		32.5 (0.3)	7.8 (0.1)	0.93 (0.04)	9.1 (0.1)

*BO*, *Brassica oleracea*. CAU, chelating activity unit. EtOH, ethanolic extract. GA, gallic acid. HP, protein hydrolysate. ABTS, 2,2′-azino-bis3-ethylbenzothiazoline-6-sulfonic acid. ICC, iron-chelating capacity. IRC, iron-reducing capacity. NT, nutraceutical. TPC, total polyphenol content. Results are the means plus the SD (in parenthesis) of four replicates expressed per milligram of extract. Means with different letters (a, b, c and d) indicate significant differences (ANOVA *p* < 0.05).

**Table 4:** Dializability and antioxidant activity of NT after an in vitro digestion.

	Dialyzed		Retained	
	Blank	NT	Blank	NT
Dializability (%)		73.1 ± 2.3		
TPC (µg GA)	7.21 ± 0.23	54.5 ± 1.1 *	8.24 ± 0.4	56.8 ± 1.2 *
ABTS (µg GA)	8.96 ± 0.23	16.0 ± 0.4 *	7.05 ± 0.27	15.7 ± 0.4 *
ICC (CAU)	0.59 ± 0.03	1.44 ± 0.01 *	0.41 ± 0.02	2.48 ± 0.03 *
IRC (µg GA)	0.80 ± 0.11	11.1 ± 0.55 *	1.53 ± 0.16	8.9 ± 0.3 *

CAU, chelating activity unit. GA, gallic acid. ICC, iron-chelating capacity. IRC, iron-reducing capacity. NT, nutraceutical. TPC, total polyphenol content. Results are the means plus the SD (in parenthesis) of four replicates expressed per milligram of sample. (*) Statistical significance compared to the blank according to Student’s *t*-test (*p* < 0.01).

**Table 5:** Identification of bioactive compounds in the nutraceutical.

RT	MS	COMPOUND	MF [H^−^]	% FIT	F1	F2	F3
0.95	313.0712	Pectolinarigenin	C_17_H_13_O_6_	96.65	225.0631	180.0354	120.0542
1.22	299.0403	Mumefural	C_12_H_11_O_9_	98.65	240.0316	225.069	220.0431
2.17	379.0818	Diphyllin	C_21_H_15_O_7_	34.36	293.0519	280.1175	105.0269
2.41	471.0927	Epigallocatechin (^1^)	C_23_H_19_O_11_	99.45	331.0149	253.0714	209.0065
2.67	471.108	Rubialatin A	C_27_H_19_O_8_	69.13	347.0891	293.041	267.1032
2.69	361.0923	Crotepoxide	C_18_H_17_O_8_	99.94	220.0375	159.0295	119.0415
2.70	505.171	Prunioside A	C_25_H_29_O_11_	93.83	347.0891	293.041	267.1032
3.57	305.066	(+)-Gallocatechin	C_15_H_13_O_7_	99.1	267.0891	239.0623	221.0522
3.78	465.1397	Curculigoside A	C_22_H_25_O_11_	99.5	347.0685	331.0488	253.0787
4.01	167.1553	Cimifugin (^2^)	C_22_H_27_O_11_	99.33	227.0043	220.0349	209.0298
4.16	583.2027	Lucidumoside C	C_27_H_35_O_14_	89.84	301.1084	262.0572	220.0384
4.19	551.1553	Cucumerin B	C_29_H_27_O_11_	2.85	315.1268	294.0228	262.0588
4.33	553.1557	Ligustrosidic acid	C_25_H_29_O_14_	99.07	294.0379	267.0679	253.0775
4.35	553.1557	Marinoid D	C_25_H_29_O_14_	99.07	317.038	267.0788	250.0633
4.65	445.1135	Glycitin	C_22_H_21_O_10_	1.49	317.0562	267.0958	250.0111
4.68	771.1773	Quercetin (^3^)	C_36_H_35_O_19_	8.75	267.0869	253.084	227.0035
4.72	467.1495	Dichamanetin	C_29_H_23_O_6_	10.39	253.0823	239.0681	195.0144
5.73	401.1236	Nobiletin	C_21_H_21_O_8_	91.69	347.0704	253.0765	239.0632
6.12	709.2285	Moracenin D	C_40_H_37_O_12_	38.63	668.2699	267.0976	239.0707
6.92	551.2856	Lokundjoside	C_29_H_43_O_10_	84.27	287.1523	267.1325	165.0612
6.95	389.1236	Populin/Populoside	C_20_H_21_O_8_	84.36	301.1513	220.0353	195.0285
7.34	323.1495	Chamissonolide	C_17_H_23_O_6_	99.75	309.135	262.0421	221.0459
7.87	251.0919	Nothoapiole	C_13_H_15_O_5_	n/a	220.036	207.0571	158.9771
8.32	753.2242	Catharticin	C_34_H_41_O_19_	15.22	331.0123	253.071	239.0514
8.53	325.1076	Eucalyptin	C_19_H_17_O_5_	94.85	299.1306	267.0681	220.025
8.81	619.2391	Hydrangenoside A	C_31_H_39_O_13_	54.88	556.2574	262.052	220.054
9.86	339.1232	8-Prenylnaringenin	C_20_H_19_O_5_	98.19	339.1318	239.0277	105.0285
11.36	533.3631	Pyrohyperforin	C_35_H_49_O_4_	16.01	400.2627	301.1781	159.0122
12.91	433.2015	Nesodine	C_27_H_29_O_5_	25.23	317.0427	267.1048	239.073
14.31	337.2379	Kirenol	C_20_H_33_O_4_	n/a	323.22	136.0556	119.0428
15.19	339.2535	Persealide	C_20_H_35_O_4_	n/a	325.2375	207.0567	159.0379

F1, F2, and F3: fragments. MF, molecular formula. MS, mass. RT, retention time. n/a, not applicable. (^1^) Epigallocatechin-O-(3-O-methyl) gallate. (^2^) Cimifugin ß-D-glucopyranoside. (^3^) Quercetin 3-O-a-6‴-caffeoylglucosyl-ß-1.2-rhamnoside.

**Table 6:** Effect of tumor development and nutritional or physical exercise interventions on organ weights (g) and colon length (cm).

	CT−	CT+	NT	NT + Ex
Liver	0.798 ± 0.026 ^a^	0.863 ± 0.026 ^ab^	0.868 ± 0.014 ^ab^	0.886 ± 0.017 ^b^
Kidneys	0.108 ± 0.002 ^a^	0.109 ± 0.002 ^a^	0.113 ± 0.002 ^a^	0.109 ± 0.002 ^a^
Heart	0.113 ± 0.002 ^a^	0.105 ± 0.002 ^a^	0.109 ± 0.002 ^a^	0.107 ± 0.003 ^a^
Spleen	0.071 ± 0.001 ^a^	0.165 ± 0.017 ^b^	0.132 ± 0.009 ^b^	0.150 ± 0.016 ^b^
Plantaris	0.013 ± 0.000 ^a^	0.012 ± 0.001 ^a^	0.013 ± 0.000 ^a^	0.013 ± 0.000 ^a^
Cecum	0.096 ± 0.005 ^a^	0.103 ± 0.004 ^a^	0.099 ± 0.002 ^a^	0.096 ± 0.004 ^a^
Gastrocnemius	0.095 ± 0.003 ^a^	0.095 ± 0.002 ^a^	0.103 ± 0.002 ^a^	0.105 ± 0.003 ^a^
Colon	0.119 ± 0.015 ^a^	0.338 ± 0.031 ^c^	0.248 ± 0.022 ^b^	0.262 ± 0.009 ^bc^
Colon length	6.88 ± 0.18 ^b^	5.99 ± 0.28 ^a^	6.28 ± 0.15 ^ab^	6.08 ± 0.13 ^a^

CT−, healthy animals fed the control diet. CT+, tumor-induced animals fed the control diet. NT, tumor-induced animals fed the control diet supplemented with the nutraceutical. NT + Ex, tumor-induced animals fed the control diet supplemented with the nutraceutical combined with the exercise protocol. Results are expressed as the means plus the SD (parenthesis) of 15 replicates. Means values with different letters (a, b, and c) represent significant differences (ANOVA, *p* < 0.05).

**Table 7:** Gene expression levels in the colon of mice at the end of the experimental period.

	CT−	CT+	NT	NT + Ex
*cat*	1.00 ± 0.18 ^b^	0.77 ± 0.08 ^ab^	0.93 ± 0.04 ^ab^	0.75 ± 0.04 ^a^
*gpx2*	1.00 ± 0.12 ^b^	0.64 ± 0.08 ^a^	1.21 ± 0.09 ^b^	1.05 ± 0.04 ^b^
*sod1*	1.00 ± 0.16 ^c^	0.38 ± 0.05 ^a^	0.86 ± 0.06 ^bc^	0.72 ± 0.03 ^b^
*nqo1*	1.00 ± 0.18 ^a^	0.79 ± 0.14 ^a^	2.36 ± 0.11 ^b^	2.23 ± 0.11 ^b^
*gsta1*	1.00 ± 0.14 ^c^	0.65 ± 0.08 ^b^	0.72 ± 0.08 ^b^	0.27 ± 0.02 ^a^
*cadm1*	1.00 ± 0.16 ^b^	1.00 ± 0.14 ^b^	0.75 ± 0.03 ^a^	0.67 ± 0.03 ^a^
*cdc42*	1.00 ± 0.15 ^b^	1.23 ± 0.23 ^b^	0.64 ± 0.04 ^a^	0.55 ± 0.01 ^a^
*IL-1ß*	1.00 ± 0.11 ^a^	1.73 ± 0.17 ^b^	1.56 ± 0.18 ^b^	1.29 ± 0.09 ^ab^
*IL-6*	1.00 ± 0.21 ^c^	0.54 ± 0.09 ^b^	0.39 ± 0.03 ^ab^	0.18 ± 0.01 ^a^
*pik3cd*	1.00 ± 0.09 ^b^	2.64 ± 0.28 ^c^	0.70 ± 0.07 ^ab^	0.52 ± 0.04 ^a^
*slc20*	1.00 ± 0.09 ^a^	4.49 ± 0.40 ^b^	0.97 ± 0.08 ^a^	0.70 ± 0.03 ^a^

CT−, healthy animals fed the control diet. CT+, tumor-induced animals fed the control diet. NT, tumor-induced animals fed the control diet supplemented with the nutraceutical. NT + Ex, tumor-induced animals fed the control diet supplemented with the nutraceutical combined with the exercise protocol. Results are expressed as means plus the SD (parenthesis) of 15 replicates. Mean values with different letters (a, b, and c) represent significant differences (ANOVA, *p* < 0.05).

## Data Availability

Data are available upon request due to time limitations.
